# Ceruminous Gland Tumors in Canines and Felines: A Scoping Review

**DOI:** 10.3390/ani15081138

**Published:** 2025-04-15

**Authors:** Tiruvilvamala Ramesh Lavanya, Pavan Kumar, Mun Keong Kok, Siew Mei Ong, Rozanaliza Radzi, Gayathri Thevi Selvarajah

**Affiliations:** 1 Faculty of Veterinary Medicine, Universiti Putra Malaysia, Serdang 43400 UPM, Selangor, Malaysia; trlava1912@gmail.com (T.R.L.);; 2 University Veterinary Hospital, Faculty of Veterinary Medicine, Universiti Putra Malaysia, Serdang 43400 UPM, Selangor, Malaysia; 3 Institute of Tropical Agriculture and Food Security, Universiti Putra Malaysia, Serdang 43400 UPM, Selangor, Malaysia; 4 College of Veterinary Science, Guru Angad Dev Veterinary and Animal Sciences University, Ludhiana 141004, Punjab, India; 5 CanRes MAKNA Laboratory, Institute of Bioscience, Universiti Putra Malaysia, Serdang 43400 UPM, Selangor, Malaysia; 6 Faculty of Veterinary Medicine, Universitas Brawijaya, Malang 65146, East Java, Indonesia

**Keywords:** canine, ceruminous, ear, feline, tumor, scoping review

## Abstract

Ceruminous glands are specialized apocrine sweat glands. Neoplastic transformation of these glands is often seen in the external ear canal. Tumors arising from these glands can present a diagnostic dilemma because of their varied clinical and histological manifestations. This study was conducted as little information is currently available on these neoplasms. The present study undertakes a scoping review of research on canine and feline CGTs according to the Preferred Reporting Items for Systematic Reviews and Meta-Analysis (PRISMA) guidelines for scoping reviews on three databases (NCBI-PubMed, Scopus and ScienceDirect) from 1980 to 2023 (43 years) to determine the extent of the existing literature on its clinicopathological characteristics, overall prognosis, survival rates, and biomarker studies. Seventeen canine and nineteen feline publications that met the inclusion criteria were analyzed. Eleven canine and twelve feline unpublished cases of CGTs managed in Malaysia were also reviewed. Our study concluded surgical excision as part of the treatment for this neoplasia may lengthen animals’ survival period and produce a satisfactory quality of life; however, a substantial risk of complications, especially after aggressive surgical excision, exists.

## Simple Summary

Ceruminous gland tumors (CGTs) account for the most common aural tumors in small animal populations. Despite this, scarce information exists on its occurrence, prevalence, potential risk factors, and outcome. In this scoping review, we comprehensively describe the literature on canine and feline CGTs in multiple contexts and explore whether the literature is amenable to systematic synthesis. In addition, the authors established that curative-intent surgical resection provided the greatest potential for its resolution and emphasized the need for future prospective, multi-institutional studies.

## 1. Introduction

Aural tumors account for a minor portion of ear conditions, although companion animals frequently present with ear diseases [1]. Canine and feline ear canal tumors are relatively scarce in comparison to integumentary neoplasms [2]. However, aural tumors often have a good prognosis if identified and promptly treated at an early stage of the disease [3].

Ceruminous glands are modified apocrine sweat glands [4,5]. Neoplastic transformation of these glands is mostly seen in the external ear canal [6,7]. Ceruminous gland tumors (CGTs) account for 6–7% of all feline and 1% of all canine skin tumors [4]. These neoplasms are histologically sub-classified as adenomas, adenocarcinomas, and simple, mixed, or complex CGTs [8,9]. Ceruminous gland adenoma (CGA) is the most prevalent benign ear canal tumor in dogs, while the malignant counterpart, ceruminous gland adenocarcinoma (CGAc), is frequently observed in cats as well as dogs [2,10,11]. Animals with a history of chronic long-standing inflammation, pruritus, otorrhea or recurring ear infections are more likely to be at an elevated risk of developing these tumors [2,5,12,13,14,15].

Depending on the location of the tumor, surgical approaches like partial or total pinnectomy, lateral ear canal resection (LECR), vertical ear canal ablation (VECA) for tumors contained within the vertical ear canal, or a more aggressive total ear canal ablation with a lateral bulla osteotomy (TECA-LBO) for malignant tumors to achieve clean margins can be adopted [16,17,18]. However, several complications are associated with TECA-LBO surgeries, some occasionally serious [19,20,21]. They include mild wound healing impairments like surgical site swelling and infection, hemorrhage, discharge, cellulitis, dehiscence, incisional abscess, or hematoma. More severe complications comprise pinnal necrosis or deafness as well as nerve damage leading to neurologic dysfunction [10,16,19,20,21,22,23]. Following the successful removal of benign tumors, the prognosis is generally optimistic, while in the case of malignant tumors, there exists a potential for local recurrence or metastasis [10,18].

The lack of a standardized diagnostic protocol, limited awareness among pet owners, and the diverse clinical presentation of CGTs contribute to delayed diagnosis and suboptimal treatment outcomes. At present, limited information is available on the impact of demographic and socioeconomic factors on the diagnosis, treatment, and overall survival of canines and felines with CGTs. A scoping review was conducted to systematically map the research conducted in this area and identify existing knowledge gaps. This review aimed to comprehensively explore the literature on clinicopathological characteristics, overall prognosis, survival rates, and biomarker studies on canine and feline CGTs.

## 2. Materials and Methods

### 2.1. Protocol

A methodology was defined prior to conducting the review and a protocol was outlined. This review followed the framework proposed by the Preferred Reporting of Items for Systematic Reviews and Meta-Analyses extension for Scoping Reviews (PRISMA-ScR) [24]. The review protocol used was not registered.

### 2.2. Search Strategy

Online/electronic bibliographic databases PubMed, Scopus, and ScienceDirect were explored for published literature from January 1980 until December 2023. Advanced search strings used for canine studies included: “canine” OR “dog” AND “ceruminous” OR “ceruminous gland tumor” OR “ceruminous gland tumour” OR “ceruminous gland neoplasm” and similarly, “feline” OR “cat” AND “ceruminous” OR “ceruminous gland tumor” OR “ceruminous gland tumour” OR “ceruminous gland neoplasm” for feline studies. The last search for the above was run on 1 January 2024 with no language restrictions. The title, authors’ names, abstract, journal name, publication year, cited by, and DOI of the records were exported to a Microsoft Excel spreadsheet.

### 2.3. Eligibility Criteria and Study Selection

Following the elimination of duplicates, retrieved records were screened based on their title and abstract by two review authors. Subsequently, the full-text studies were evaluated according to the eligibility criteria. To be included, publications had to be case reports or series, original research articles, or retrospective studies on canine and/or feline ceruminous gland tumors. Eligibility was assessed following PCC framework (Population: canine and feline with CGTs; Concept: clinicopathological characteristics, treatment protocol, outcome, survival, biomarker studies; Context: N/A). Studies were excluded for the following reasons: duplicates, non-English papers, abstracts for which the full-text records were unavailable, other tumors affecting the ears, species apart from canine and feline, books, or book chapters. Manuscripts on non-tumor conditions such as ceruminous otitis, Malassezia otitis, and otitis externa were also excluded.

### 2.4. Data Extraction and Analysis

Additional data, if available, were extracted from the eligible records: type of publication, country, number of animals studied, signalment and clinical presentation, diagnostic methods, mode of treatment, diagnosis, clinical outcome, evidence of metastasis, recurrence of the condition post-treatment, survival data, and biomarker studies. Subsequently, descriptive analysis was performed, and data were presented in frequency and percentages.

## 3. Results

### 3.1. Outcome of Case Selection

**Figure 1:**
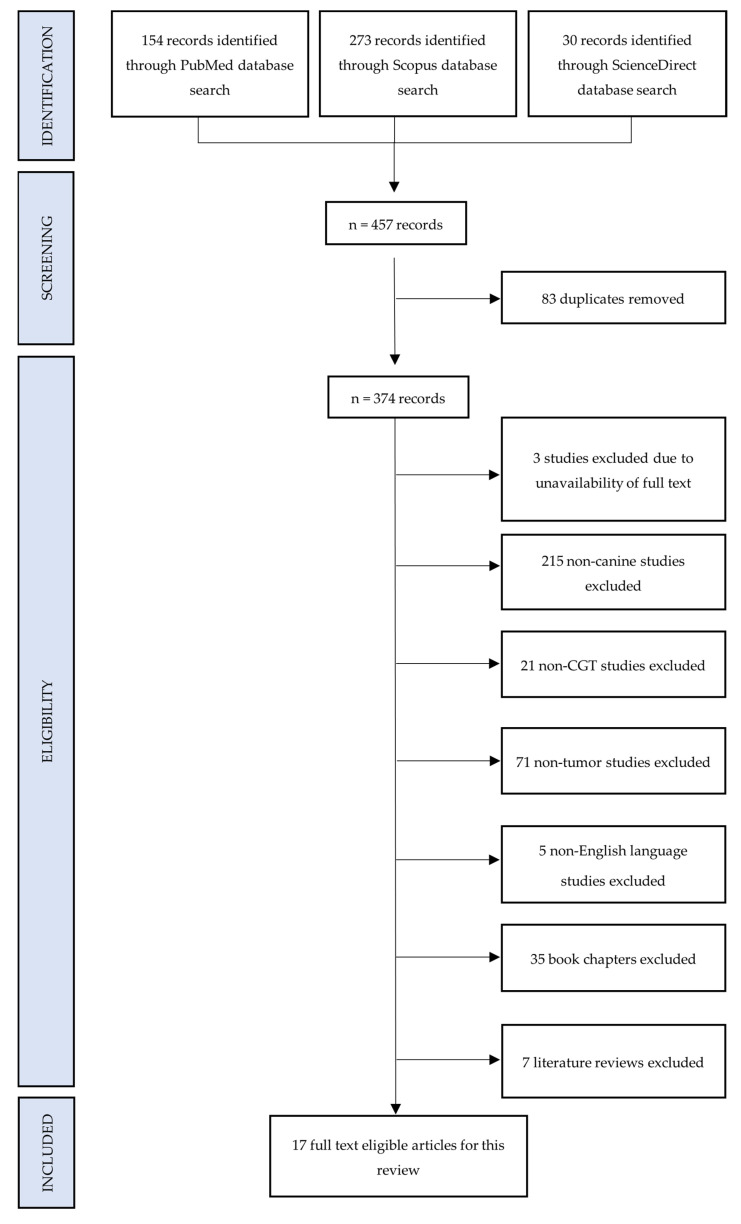
Preferred reporting items for systematic reviews and meta-analyses for scoping reviews flowchart of selected canine studies.

Canine: The methodology adopted for this scoping review adhering to the PRISMA-ScR guidelines, as illustrated in Figure 1, presents a flowchart outlining the inclusion and exclusion criteria applied to past records. Comprehensive searches were conducted on three prominent databases, namely PubMed, Scopus, and ScienceDirect utilizing advanced search strings.

The initial search yielded a total of 457 journal articles. Following this, 83 duplicate articles were identified and subsequently removed, resulting in a refined dataset of 374 unique articles. The next step involved a meticulous evaluation of each article against predefined inclusion and exclusion criteria as mentioned in Section 2.3. Out of the 374 articles, three were excluded due to the unavailability of full-text manuscripts. Further exclusions were made based on the nature of the studies. A total of 215 articles were unrelated to canines and consequently excluded. Additionally, 21 articles were eliminated as they did not pertain to CGTs, and 71 more were excluded for not focusing on tumor-related aspects. Language constraints were also considered, leading to the exclusion of five studies not presented in English language. Furthermore, thirty-five book chapters and seven literature review papers deemed irrelevant to this review were excluded as well. Thus, the review was conducted on 17 records [13,25,26,27,28,29,30,31,32,33,34,35,36,37,38,39,40]. Seven publications were from the United States of America, three from Japan, two from India, and the rest from Bulgaria, Italy, Korea, Netherlands and Portugal. These studies were then subjected to a comprehensive analysis to extract relevant data, forming the basis for the findings and conclusions presented in this study.

**Figure 2:**
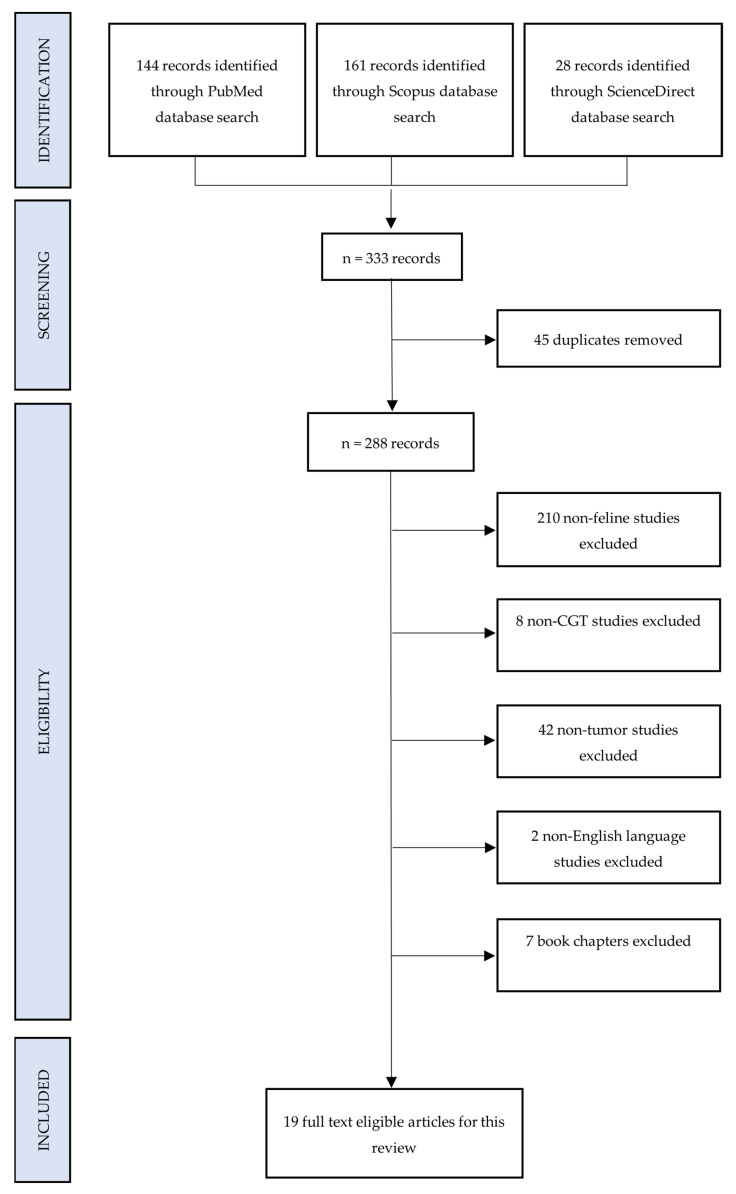
Preferred reporting items for systematic reviews and meta-analyses for scoping reviews flowchart of selected feline studies.

Feline: The methodology adopted for this scoping review adhering to PRISMA-ScR guidelines, as illustrated in Figure 2, presents a flowchart outlining the inclusion and exclusion criteria applied to past records. Comprehensive searches were conducted on three prominent databases, namely PubMed, Scopus, and ScienceDirect, utilizing advanced search strings.

The initial search yielded a total of 333 journal articles. Following this, 45 duplicate articles were identified and subsequently removed, resulting in a refined dataset of 288 unique articles. The next step involved a meticulous evaluation of each article against predefined inclusion and exclusion criteria as mentioned in Section 2.3. Two hundred and ten articles were unrelated to felines and thus excluded. Additionally, eight studies were eliminated as they did not pertain to CGTs, and forty-two more were excluded for not focusing on tumor-related aspects. Language constraints were also considered, leading to the exclusion of two studies not presented in English language. Furthermore, seven book chapters deemed irrelevant to this review were excluded as well. Thus, the review was conducted on 19 eligible records [13,14,26,28,29,30,31,34,41,42,43,44,45,46,47,48,49,50,51]. Thirteen of these publications were from the United States of America, two from the United Kingdom and the rest from Egypt, Italy, Japan, and Libya. These studies were then subjected to a comprehensive analysis to extract relevant data, forming the basis for the findings and conclusions presented in this study. This transparent and systematic approach adopted throughout the process ensured the reliability and reproducibility of the results obtained.

### 3.2. Summary of Canine and Feline Ceruminous Gland Tumor Cases

Data from the selected studies were pooled to extract information on signalment, clinicopathologic characteristics, treatment approaches, survival, and biomarker studies; some of which have been summarized in Table A1, Table A2 and Table A3.

(a) Signalment and Clinical Presentation

Canine: One hundred and seventy canine cases were under consideration among the selected seventeen studies, a breakdown of which is given in Table A1 and Table A3. Among these, 57 were CGA, including two complex CGA [25,26]; 108 were CGAc, including two simple and two complex CGAc [27]. Three cases were diagnosed as mixed CGAc ([21] (n = 2); [28] (n = 1)) and two as mixed CGT (unspecified) [26]. One case had a differential of either an adenoma or an adenocarcinoma [26]. Folk and colleagues [29] studied eight CGA and five CGAc, but no segregation was provided for cat and dog specimens. Eight studies specified the breed, i.e., among the 170 cases, the breed was known for 33 canines only. Nine dogs were mixed breeds, eight were Cocker Spaniels (American Cocker Spaniel-1; English Cocker Spaniel-5, unspecified-2), four were Poodles, three were Shih Tzu’s and one each of Beagle, Dachshund, Doberman, English Mastiff, English Springer Spaniel, French Bulldog, Pug, Spitz, and Terrier. The age of presentation was specified in 65% (n = 11/17) of studies. The mean age was reported as 10.44 years for CGA and 9.1 for CGAc [26], 9 years for CGA, and 11 for CGAc [28], 10 years [30] and 9.55 ± 3.3 years [31]. The remaining studies reported a mean age of 8.9 years with the youngest aged 4 years and the oldest aged 13 years [25,32,33,35,36,37,39]. Gender information was available in 59% (n = 10/17) of studies. Male canines were overrepresented with seventeen intact and six castrated males. Intact females were twelve and spayed were six yielding a total male-to-female ratio of 1.28:1.

An association between chronic otitis externa and CGTs has been deduced [2,5] which was observed to be one of the most common presenting conditions in dogs [13,25,26,28,29,32,33,34]. Data on unilateral or bilateral CGTs were available in nine papers [25,30,31,32,33,34,35,36,37]. Most dogs were affected unilaterally (n = 33/34 cases) by the tumor (unilateral or bilateral data available for 34 cases). Although, aural neoplasia is often said to occur unilaterally [52], Spivack and colleagues [34] reported a dog with CGA in the right ear and a CGAc in the left ear (n = 1/34).

Feline: Two hundred and forty-eight feline cases were under consideration among the selected nineteen studies, a breakdown of which is given in Table A2 and Table A4. Among these, 120 were CGA and 128 were CGAc. One study did not specify the number of cases involved [29]. Nine studies specified the breed, i.e., among the 248 cases, the breed was known for 88 felines only. Forty-eight were domestic shorthair cats, fifteen were domestic medium hair, eight were domestic long hair, eight were Persians, four were Egyptian Maus, and an Abyssinian, American short hair, Himalayan, Persian mix and a Siamese each was reported. The age of presentation was specified in 63% (n = 12/19) of studies. The mean age was reported as 10 ± 2.0 years [41], 12.6 years [30], and 9.8 ± 3.0 years [31]. Moreover, Moisan and Watson [28] reported the mean age as 8.5 years and 11 years for CGA and CGAc, respectively. Similarly, van der Gaag [26] reported the mean age of CGA as 9.37 years and CGAc as 8.66 years. The remaining studies reported a mean age of 9.125 years with the youngest aged 5 years and the oldest aged 18 years [14,44,46,47,48,49,50]. Gender information was available in 58% (n = 11/19) of studies. Male felines were overrepresented with 11 intact and 55 castrated males. Intact females were 12 and spayed were 29 yielding a total male-to-female ratio of 1.6:1.

Otitis externa/inflammatory changes were observed in 68% (n = 13/19) of publications [14,28,29,30,34,41,42,43,44,45,46,47,48]. Data on unilateral or bilateral CGTs was available in 12 papers [14,26,30,41,43,44,45,46,47,48,49,50]. The tumor was reported to occur unilaterally (n = 42/103 cases) (7 CGA, 35 CGAc) [30,41,43,44,46,47,49,50] but a substantial number of these publications also reported a bilateral occurrence (n = 61/103 cases) (51 CGA, 10 CGAc) [14,26,30,43,44,45,48,49].

(b) Treatment Options, Clinical Outcome and Survival

Canine: Aggressive surgical excision employing total ear canal ablation (TECA/combined with lateral bulla osteotomy, LBO) has been described as the best course of action for CGTs [17,18,21,52]. Surgical management was achieved via this approach in 30% (n = 5/17) of papers [[29] (unspecified), [30] (three CGAc, species unspecified), [32] (one CGAc), [34] (five CGA, seven CGAc), and [35] (one CGAc)]. Another 24% (n = 4/17) of papers mentioned treatment and sample collection through surgical excision/resection of the tumor mass [[36] (one CGA), [37] (one CGA), [38] (one CGAc), [39] (eight CGA, and eight CGAc)]. Kokila and colleagues [37] (one CGA) also mentioned the use of electrocautery to achieve the same. Moreover, 24% (n = 4/17) of papers used surgical biopsies/specimens from the histopathology archives to conduct their study [[26] (nine CGA, one complex CGA, five CGAc, one mixed CGT, one CGA/CGAc), [27] (two simple CGAc, two complex CGAc, two mixed CGAc), [28] (twenty-three CGA, thirty-eight CGAc, one mixed CGT), [40] (three CGAc)]. On the other hand, Usui and peers [25] achieved a complete cure using the alternate approach of excision using a diode laser followed by repeated cleansing of the ear canal and tympanic cavity using a video otoscope. Takagi and associates [35] initially performed a total ear canal ablation on a CGAc; following its recurrence, they employed high-temperature hyperthermia (HTH) treatment to achieve satisfactory results. Video-otoscopic aided biopsy and carbon-dioxide (CO_2_) laser ablation yielding low complication and recurrence rates was a viable alternative therapy [31] (four CGA, seven CGAc). Five of nine dogs with incompletely resected CGAc were subjected to adjuvant radiation/radiotherapy (RT). Megavoltage irradiation was used in four dogs while orthovoltage irradiation was used in one dog (no involvement of deeper structures). A 48 Gy radiation dose was given over 4 weeks (Monday, Wednesday, Friday schedule) at 4 Gy fractions [30]. London and colleagues [13] reported the use of surgical excision, RT as well as chemotherapy; however, the specific treatment employed for tumors of relevance to this study is unclear. In a single instance, no surgical intervention was carried out owing to the animal’s poor health; instead, euthanasia and a postmortem were performed [33].

Histopathology was the only mode of confirmative diagnosis for all the mentioned samples of CGTs. Tumors in this review were either classified as adenomas or adenocarcinomas and additionally as simple, complex, or mixed subtypes (Table A3). Mitotic figures (MF)/mitotic activity was mentioned in 35% (6/17) of publications. In a complex CGA, few MF were observed [25]; whilst, moderate mitotic activity was noted in a CGAc [33]. The frequency of mitosis was a criterion for the histopathological classification of samples for Simeonov’s [39] study. Théon and peers [30] reported up to seven mitotic figures per high power field (MF/HPF) in canine CGAc. Furthermore, he also described the possible correlation between the mitotic rate and degree of differentiation, i.e., well-differentiated tumors had rare MF, whereas, poorly differentiated tumors had 3–5 MF/HPF. Similarly, Moisan and Watson [28] reported up to 10 MF/HPF in CGAc and <1 MF/HPF in CGA. Frequent MF up to 24 MF/10 HPF was observed as well [32].

Limited clinical outcome and survival data were available. Recurrence of the tumor was reported in 24% (n = 4/17) of publications [[30] (two dogs), [31] (two cases, species unspecified), [32] (one dog), [35] (one dog)]. Ceruminous gland tumors seldom metastasize beyond the regional lymph nodes [9]. However, they may exhibit local invasiveness, with rare instances of metastasis to regional lymph nodes, salivary glands, lungs, and distant viscera [22,33,53].

One dog died due to respiratory distress of unknown cause; however, no evidence of pulmonary metastasis was observed in the thoracic radiograph. In the same case report, massive metastatic foci originating from the ceruminous gland was observed in the excised parotid lymph node on histopathology [32]. Among 16 canine CGT (eight CGA, eight CGAc), three CGAc exhibited metastases in the regional lymph nodes (unspecified), confirmed by fine-needle aspiration cytology/biopsy (FNAC/FNAB/FNB) in a Poodle, an English Cocker Spaniel, and a mixed-breed dog [39]. Romanucci and co-workers [33] euthanized a dog due to poor prognosis which was followed by conducting a post-mortem to reach a conclusive diagnosis of CGAc based on histopathology. Pulmonary metastases, and diffuse gross enlargement with metastatic infiltration of right submandibular, parotid, cervical, retropharyngeal, and prescapular lymph nodes were recorded. Regional lymph node metastasis and recurrence were recorded in London and colleagues’ [13] study; however, details on whether the tumors were CGTs are unclear. In the same study, out of the twenty-three dogs diagnosed with CGAc, three succumbed to the consequences of the tumor, compared to four out of eight dogs with squamous cell carcinoma. Another study reported two dogs had a recurrence of the CGAc: one was surgically treated with TECA while the other was treated with TECA combined with megavoltage irradiation (previously treated with orthovoltage irradiation). After 20 months of survival, the latter dog developed pulmonary metastases [30]. Additionally, one animal each (species unspecified) had regional lymph node metastasis and metastasis to the intra-abdominal organs. Following CO_2_ laser ablation of the tumors, limited post-operative complications were observed with only four dogs (n = 4/11) developing otitis during the 30-day post-surgical period [31]. Additionally, only two CGTs (one CGA and CGAc each) (species unspecified) recurred in his study.

Feline: Total ear canal ablation (TECA/combined with LBO) was the most used surgical treatment approach in 37% (n = 7/19) of papers [[29] (unspecified), [30] (3 CGAc, species unspecified), [34] (3 CGA, 1 CGAc), [41] (3 CGA, 7 CGAc), [43] (18 CGAc), [45] (4 CGA, unclear), and [49] (7 CGAc)]. Conservative resection on three occasions followed by complete surgical removal of the tumor was adopted in a cat with CGAc [14]. Whereas, an exploratory surgery of the ear canal combined with a lateral bulla osteotomy was performed in another [50]. Pinnectomy in two cats (CGA) was mentioned by Loft et al. [45] while Pavletic [46] adopted partial vertical ear canal resection for two cats (CGA). The improved cosmetic appearance of the pinna was attained with the use of a modified TECA technique (ventrally based advancement flap for closure of TECA) (two CGA, two CGAc) [44]. Normal ear carriage was attained with one other modified technique, namely the subtotal ear canal ablation in a single cat with a CGA [47]. Another plausible alternative therapy was video-otoscopic aided biopsy and CO_2_ laser ablation, which yielded low complication and recurrence rates [31]. Corriveau [48] also described the employment of a CO_2_ laser in the treatment of a case of CGA. Six of nine cats with incompletely resected CGAc were subjected to adjuvant RT (megavoltage irradiation was used in these six cats) [30]. Similar to the results in canine, London et al. [13] mentioned the use of surgical excision, RT as well as chemotherapy; however, the specific treatment employed for tumors of relevance to this study was unclear.

Confirmative diagnosis for the samples of CGTs was achieved via histopathology. Fifteen per cent (n = 3/19) of the papers used surgical biopsies/specimens from the histopathology archives to conduct their study [[26] (eight CGA, three CGAc), [28] (19 CGA, 43 CGAc), [51] (two CGAc)]. Ultrasonography [41] and FNAC/FNAB/FNB [42] were also used to diagnose feline CGTs. However, histopathologic confirmation was recommended.

Mitotic activity/mitotic figures (MF) were reported in 31% (n = 6/19) of papers. The results were similar to canines wherein adenocarcinomas exhibited higher mitotic activity than adenomas [14]. Ceruminous gland adenomas and CGAc with few and several MF, respectively, were noted by Abdelgalil and Mohammed [41]. No MF (CGA) and frequent mitosis (CGAc) were observed by De Lorenzi and colleagues [42]. Théon et al. [30] reported up to five mitotic figures per high power field (MF/HPF) in feline CGTs. Furthermore, he also described the possible correlation between the mitotic rate and degree of differentiation, i.e., well-differentiated tumors had up to 2 MF/HPF, whereas poorly differentiated tumors had 4–7 MF/HPF. Likewise, Moisan and Watson [28] observed up to 10 MF/HPF in CGAc and <1 MF/HPF in CGA. The mitotic index (MI) was also considered a potential prognosticator for CGAc [43], wherein cats with MI ≤ 2 had a significantly longer survival time than those with MI ≥ 3 (MI was the mean number of mitotic figures per ×40 HPF).

Information regarding the clinical outcome and survival was minimal. Nine studies (n = 9/19) [13,30,31,43,44,46,47,48,49] provided follow-up data. Three out of seven cats with CGAc died three, four, and six months postoperatively (TECA/LBO); additionally, two of these cats were affected bilaterally (6-month follow-up period) [49]. On the other hand, the median survival time for 12 cats treated with TECA for CGAc was 50.3 months [43]. The median survival time of 18 cats with CGAc was >49 months compared to 3.8 months for cats with squamous cell carcinoma [13]. Furthermore, four cats with follow-up periods ranging from 2 to 16 months reported minimal complications and overall satisfactory owner assessment following modified TECA [44]. In both the affected cats, Pavletic [46] mentioned a disease-free interval of 10 and 12 months, respectively. Théon et al. [30] mentioned a follow-up duration ranging from 5 to 66 months; however, this was the combined result for canines and felines in his study. He further mentioned no recurrence in six animals; however, two among these died due to unrelated causes, while four animals lived without evidence of tumor recurrence. Similarly, a follow-up duration (common for canine and feline CGTs) of 16.5 months (3–50 months) was described by Pieper et al. [31]. A cat treated with subtotal ear canal ablation for CGA with a follow-up period of eight months had satisfactory results and no ear problems [47]. Corriveau [48] conducted regular reviews of a cat at 1 week, 17 days, 24 days, 36 days, and 2 months post-surgery. He also followed up with the cat for a total period of 2.5 years and confirmed no recurrence of the lasered masses during this period.

Ceruminous gland adenocarcinomas are more likely to occur in felines than canines [2,4,11] and their metastatic potential has been recorded [13,18,54,55]. Marked lymphatic metastasis was reported only in three feline CGAc (n = 3/7) [41]. Regional lymph node metastasis and recurrence was recorded; however, details on whether the tumors were CGTs are unclear [13].

Recurrence of the tumor was recorded in 31% (n = 6/19) of publications [[14] (one cat), [26] (one cat), [30] (two cats), [31] (two cases, species unspecified), [43] (six cats were euthanized as a result of recurrence or old age), [46] (one cat)]. After the masses were initially observed and resected (5 years ago), they recurred twice [14]. Six cats (of the 12 cats with follow-up times greater than 6 months) with CGAc were euthanized due to tumor recurrence or old age (unspecified) by Bacon et al. [43]. Despite having a disease-free interval for 12 months, a cat treated with partial vertical ear canal resection developed a CGAc in the adjacent rostro-lateral margin of the vertical ear canal 1-year post-surgery. After the mass was resected again, the cat experienced no recurrence for four months [46]. Théon and peers [30] recorded a mean progression-free survival (PFS) time of 39.5 months and a one-year PFS rate of 56% (combined results for canine and feline). However, two cats (of six cats) with CGAc had a recurrence. Compared to the original CGAc, these tumors were more anaplastic. In one cat, the recurring tumor (in the original area of the CGAc) was identified as a squamous cell carcinoma. Pieper and associates [31] mentioned the recurrence of two CGTs (an adenoma and adenocarcinoma each); but, these results were common for canine and feline. On the other hand, in a single cat, four distinct tumors were identified during the follow-up period. During the first resection, one ear had a sebaceous gland adenoma, while the other had a squamous cell carcinoma and a CGA. Eventually, a tumor recurred in the resected region, which was identified as a sebaceous gland carcinoma [26].

(c) Biomarker Studies

Canine: Among the selected studies, only a minority, constituting 24% (4/17) furnished details on IHC. Romanucci et al. [33] performed immunohistochemical staining against cytokeratins AE1 ⁄AE3, CAM5.2, vimentin, α-smooth muscle actin (α-SMA), and S100 protein. The final diagnosis of CGAc was supported by the fact that most of the neoplastic cells stained positively for CAM5.2, while the overlying epidermis was negative. This result not only substantiated the final diagnosis but also validated the efficacy of CAM5.2 in distinguishing these tumors from aural squamous cell carcinoma.

Three CGAc tissues were subjected to IHC against cytokeratins (CK7, CK14), vimentin and Bcl-2 [40]. Positive expression for cytokeratins (CK7+/CK14+) and vimentin was expressed by all three tumors, whereas two of three CGAc were immunopositive for Bcl-2. Pieper et al. [40] further inferred that the absence of CK14 and vimentin expression in CGAc could be valuable in distinguishing benign and malignant processes, especially when dealing with small biopsy specimens. Multifocal loss of CK14 and vimentin staining (myoepithelial cells) was observed in the adenocarcinomas despite all the apocrine gland and CGTs expressing CK7+/CK14+. Given that myoepithelial cells co-express CK14 and vimentin, coordinate loss of both these markers would be expected where there is a loss of myoepithelial cells of apocrine and ceruminous glands. Loss of this myoepithelial cell layer is a key event in the progression towards invasive carcinoma. But this feature alone cannot be used to diagnose a malignant process [56]. Kang et al. [32] also conducted IHC on a metastatic CGAc which exhibited strong immune positive reactions for CK7 and negative for CK 5/6.

Another reliable marker for basilar and myoepithelial cells of canine malignant apocrine and CGTs is p63 [27], a homologue of p53 [57] which was expressed by the myoepithelial cells of apocrine as well as CGTs. The findings of Saraiva et al. [27] for tumors originating from apocrine and ceruminous glands showed similarity because ceruminous glands are modified apocrine sweat glands. In cases with difficulty distinguishing myoepithelial proliferation, p63 may be useful in their classification and p63 may play a role in the oncogenesis of these tumors.

Feline: None of the selected feline publications described the employment of immunohistochemical studies for CGTs. However, Mochizuki and colleagues [51] described genetic and epigenetic alterations of p16 in feline lymphoid and non-lymphoid malignancies, wherein deletion/mutation was undetected in two feline CGAc.

### 3.3. Unpublished Malaysian Cases

**Figure 3:**
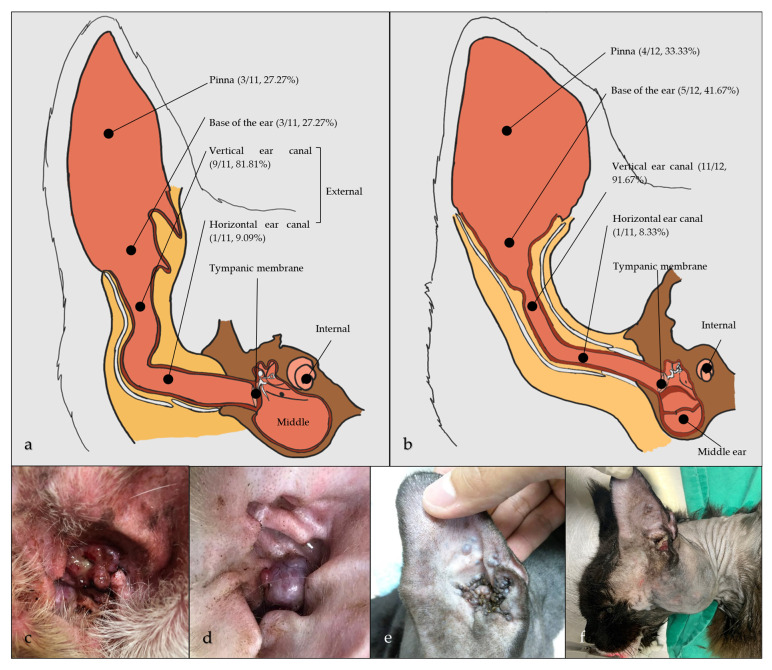
Cross-sectional schematic diagram of the: (**a**) canine external, middle, and internal ear; (**b**) feline external, middle, and internal ear indicating the relative frequency of CGT locations (adapted and redrawn from [58]); (**c**) cauliflower-like mass with serosanguineous discharge, canine case 4 (CGAc); (**d**) nodular mass with clear, waxy discharge, canine case 9 (CGAc); (**e**) bluish-grey nodules on the pinna and occluding the ear canal orifice, feline case 3 (CGA); (**f**) hard swelling at ventrolateral aspect indicating calcification-associated changes, feline case 12 (CGAc).

Eleven canine and twelve feline unpublished cases of CGTs managed in a referral veterinary hospital in Malaysia meeting the inclusion criteria were recorded. Signalment and clinical presentation of the cases are shown in Table 1 and Figure 3. Lesions were mostly observed in the vertical ear canal (Figure 3a,b). The diagnostic protocol included routine hematology and serum biochemistry, otoscopy, cytology, bacterial culture and antibiotic sensitivity, and diagnostic imaging before surgical intervention.

**Figure 4:**
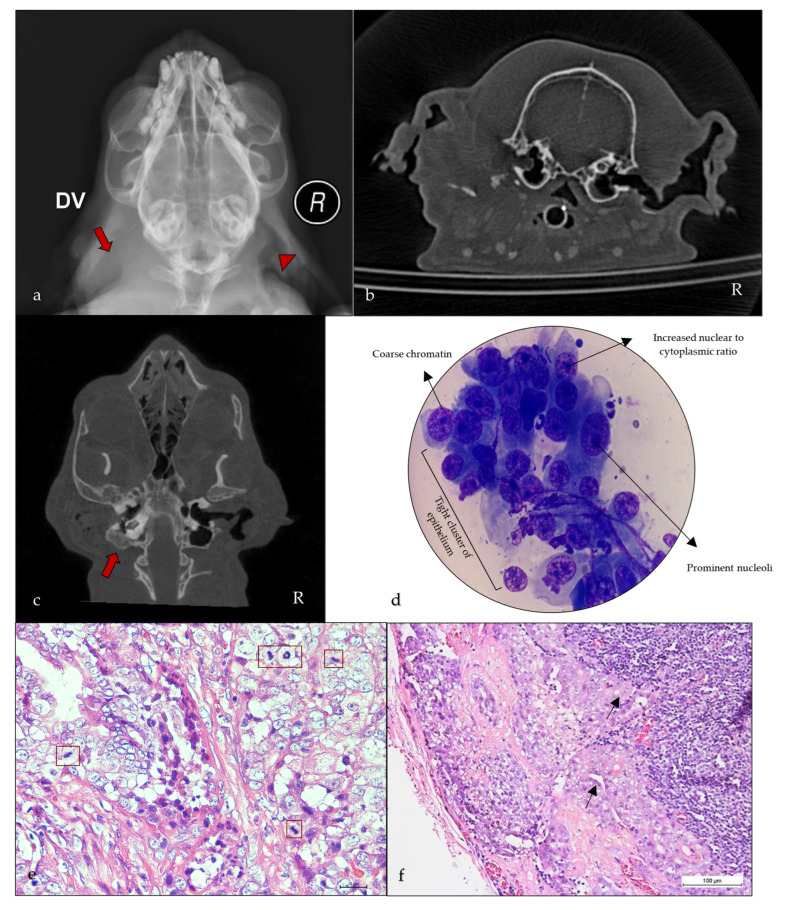
Diagnostic imaging, cytological and histological findings of canine and feline ceruminous gland tumors: (**a**) occluded left ear canal with increased soft tissue opacity (arrow) compared to apparently normal right ear canal (arrowhead), dorsoventral view (radiograph), feline case 2 (CGAc); (**b**) isodense soft tissue mass extending along the external ear canal till the tympanic membrane with mild thickening of the bulla, coronal view (CT), canine case 5 (CGAc); (**c**) complete occlusion of the left external ear canal with loss of air in the fluid-filled left tympanic cavity (arrow), axial view (CT), feline case 5 (CGA); (**d**) cytology of ear mass revealing increased nuclear-to-cytoplasmic ratio, prominent nucleoli, coarse chromatin, anisokaryosis, and anisocytosis, feline case 2; (**e**) mitotic figures (red box) in a canine CGAc, hematoxylin and eosin (40× Obj. Bar, 10 µm); (**f**) metastatic CGAc in the submandibular lymph node (black arrows indicating metastasis), feline case 2 (10× Obj. Bar, 100 µm).

Canine: Among the evaluated animals, males were overrepresented at 81.82% (n = 9/11) compared to 18.18% (n = 2/11) of females. At the time of diagnosis, the mean age was 9.6 years. Otitis was observed in 81.82% (n = 9/11) of dogs. Diagnostic skull radiography was performed in 45.45% (n = 5/11) of dogs and computed tomography (CT) in 36.36% (n = 4/11) of dogs (Figure 4b). Additionally, thoracic radiography was done in 63.63% (n = 7/11) of dogs to assess for pulmonary disease and metastasis. None of the dogs showed pulmonary disease or nodules/lesions suggestive of pulmonary metastasis or tracheobronchial lymphadenopathy.

Feline: Males were overrepresented at 75% (n = 9/12) compared to 25% (n = 3/12) females. At the time of diagnosis, the mean age was 7 years. Otitis was observed in 75% (n = 9/12) of cats. Diagnostic skull radiography was performed in 58.33% (n = 7/12) of cats and CT in 50% (n = 6/12) of cats (Figure 4a,c). Thoracic radiography was performed in 83.33% (n = 10/12) of cats. Suspicious pulmonary nodules were observed in a cat (8.33%, n = 1/12) which died three days post-TECA-LBO; however, no postmortem examination was conducted to confirm if the CGA was metastatic and the cause for death or for the possible presence of malignant tumors.

Cytology was performed in 27.27% (n = 3/11) of dogs and 33.33% (n = 4/12) of cats (Figure 4d) to achieve a tentative diagnosis. A confirmative diagnosis, however, required further diagnostics.

The majority of the cases were surgically managed with TECA-LBO, while TECA, VECA, traction, or lumpectomy were successful in the others (Table 1). Two dogs and four cats were diagnosed with CGA while the remaining nine dogs and eight cats with CGAc. All cases were confirmatively diagnosed on histopathology. Mitotic count was available for nine canine and eight feline cases (mitotic count was calculated in 10 HPFs (×40 objective; 2.37 mm^2^); it ranged from 0 to 4 (0.89 ± 1.76) in canines and 0 to 5 (1.5 ± 2.2) in felines (Figure 4e,f).

**Figure A1:**
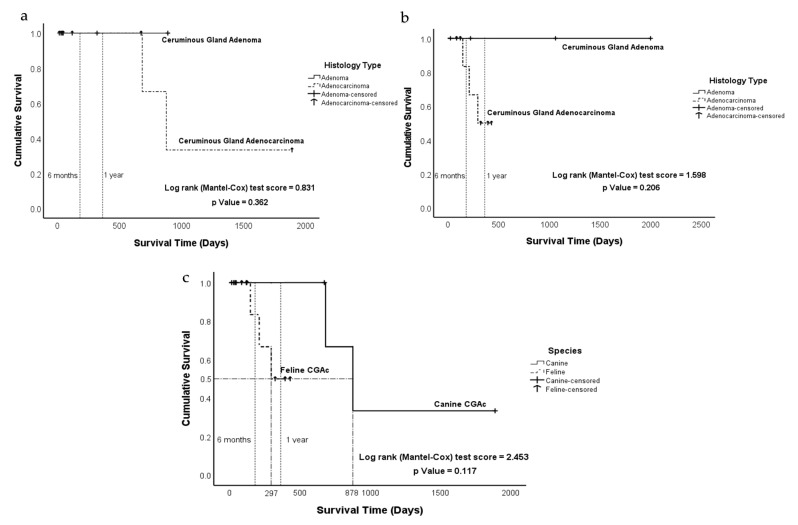
Kaplan-Meier survival curve depicting overall survival. (**a**) K-M survival curve for canine ceruminous gland tumor groups; (**b**) K-M survival curve for feline ceruminous gland tumor groups; (**c**) K-M curve for canines and felines diagnosed with ceruminous gland adenocarcinoma (CGAc).

At the end of the study period, 45.45% (n = 5/11) of dogs and 25% (n = 3/12) of cats were lost to follow-up. Two dogs (18.18%, n = 2/11) and three cats (25%, n = 3/12) died due to CGT, two dogs (18.18%, n = 2/11) and four cats (33.33%, n = 4/12) died from other causes, and one dog was euthanized due to poor quality of life. Kaplan–Meier (K-M) curves were used to compute survival analysis. Figure A1a shows no significant difference between canine histological groups (CGA vs. CGAc, *p* = 0.362) regarding survival time. Based on Figure A1b, no significant difference in survival between feline histological groups (CGA versus CGAc, *p* = 0.206) were observed. No significant difference in survival between canine and feline CGAc was determined by a log rank test (*p* = 0.117). The median survival time of dogs with CGAc was 878 days and cats with CGAc was 297 days (Figure A1c).

Evidence of metastasis was seen in 25% (n = 3/12) of cats: one to the submandibular lymph node (CGAc), one to the mandibular salivary gland, submandibular, and prescapular lymph node (CGAc), and one showed signs of pulmonary metastasis (CGA). However, one must consider the possibility of the latter cat having a concurrent malignant tumor.

During the follow-up period, recurrence of the mass was recorded in two dogs (one with CGA and CGAc each) (18.18%, n = 2/11) and three cats (25%, n = 3/12) with CGAc. Of the two dogs, one had a mass in the previously affected ear (183 days post TECA) while the other dog in the opposite (19 days post TECA-LBO). In a cat treated with chemotherapy, local regrowth was observed 49 days post-TECA-LBO, while another cat developed a small mass 39 days post-TECA-LBO. The former cat’s condition deteriorated and eventually died, and the latter was histologically diagnosed with metastatic adenocarcinomas of the ipsilateral submandibular and prescapular lymph node. The third cat developed a mass 181 days post-TECA-LBO, bilateral submandibular lymphadenopathy, neurologic complications, and eventually died 260 days post-TECA-LBO.

## 4. Discussion

Aural tumors account for 2 to 6% of all canine tumors and 1 to 2% of all feline tumors, thus emphasizing their rarity [2,23,54,59]. Put simply, despite being the most common tumor found in the canine and feline ear canal, CGTs are considered rare. One can say that most dogs and cats presented for surgical treatment often have multiple complications; additionally, the cost of diagnostics and treatment may deter owners from bringing their pets for treatment leading to underreported cases.

**Figure 5:**
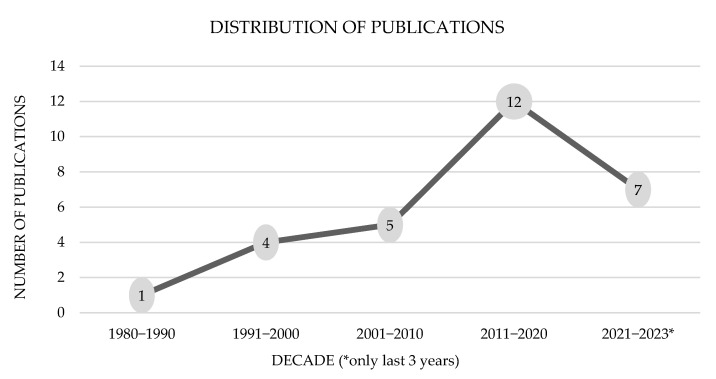
Distribution of publications across decades.

From the initial 457 canine and 333 feline studies, we excluded and selected a much smaller number to review, details of which are thoroughly explained in Figure 1 and Figure 2. To the best of our knowledge, this is the first scoping review on canine and feline CGTs. As expected, the search results of this study are in accordance with the above-mentioned rarity, i.e., this review collected and analyzed only 17 canine and 19 feline papers published between 1980 and 2023 (43 years). In addition, the authors also sought to compile pertinent data to provide a better understanding of the signalment, clinicopathologic characteristics, various treatment approaches employed, clinical outcome, survival, and biomarker studies, if any, for this particular neoplasm. A rise in the articles/case reports published in this regard was relevant in the last decade (2011–2020) with a total of 12 publications (n = 12/29; 7 same publications among canine and feline) in comparison to the previous decades (Figure 5). The United States of America (n = 13/29) accounted for the majority of these publications, with Japan (n = 3/29) coming second. For a better understanding of the etiology, clinical presentation, diagnosis, treatment, and outcome, twenty-one unpublished Malaysian cases managed at the University Veterinary Hospital were also presented in this study for the first time.

Rogers [2] suggested a cause-and-effect relationship between the so-called infection/inflammation and neoplasia. Excessive cerumen production along with ceruminous gland hyperplasia (CGH), concurrent bacterial infection, ear mite infestations, and chronic inflammation are all thought to be predisposing factors for the neoplastic transformation of these glands in dogs and cats [5,7,15]. Furthermore, it may arise from a congenital disease or senile degenerative alteration [60,61].

### 4.1. Signalment

Affected animals were generally older (late to middle-aged) [4,7] while purebred dogs (Cocker Spaniel, Shih Tzu, Poodle) [4,9,62] and domestic shorthaired cats were predisposed to developing CGTs [9]. These details were in concordance with our review. Although no definitive sex predilection has been recorded [9], a male predominance was observed in affected dogs and cats.

### 4.2. Treatment

From the data reviewed, surgery was the most effective course of treatment. Satisfactory results were achieved with: (i) TECA/combined with LBO; (ii) modified TECA; (iii) subtotal ear canal ablation; (iv) partial vertical ear canal resection; (v) carbon-dioxide (CO_2_) laser ablation; (vi) excision using a diode laser; or (vii) high-temperature hyperthermia (HTH) treatment. The greatest potential for aural neoplastic disease resolution was achieved with curative-intent surgical resection. Dogs and cats with end-stage otitis or neoplasms (especially the external ear canal) may thus benefit from TECA-LBO [43,47,63,64].

Aggressive surgical resection with a TECA-LBO is considered the most preferred approach to achieve clean margins with a better prognosis [13,17,21]; however, postoperative complications associated with this method are observed to be numerous [10,16,20,64]. Among the unpublished cases, most postoperative complications were minor and self-limiting such as serosanguineous discharge from the surgical site, suture breakdown, and wound dehiscence. Neurological complications, if any, were often temporary and more frequently recorded in felines.

Previously published data barely discuss the cosmetic results achieved with TECA in canines and felines [17,18]. Dogs with pendulous ears had little to no alteration in the cosmetic appearance [21,65,66,67]; in contrast, the postoperative deformity was frequent in dogs and cats with erect ears thus resulting in owner dissatisfaction [22,65]. McNabb and Flanders [44] performed a modified TECA wherein the erect ear carriage was preserved by creating a ventrally based advancement flap in four affected cats (CGA—two cats, CGAc—two cats). Postoperative complications with this technique were comparable to those following a standard TECA [49,64,65] and two out of four cats had complications: avascular necrosis of pinna tip/partial pinna necrosis and deafness, respectively. Overall outcome and cosmetic appearance with modified TECA resulted in owner satisfaction.

A subtotal ear canal ablation (SECA) technique for canines and felines was used by Mathews et al. [47] for the treatment of otitis externa/media or masses confined to the horizontal external ear canal. The paper involved only a single domestic shorthair cat with unilateral CGA and otitis externa that underwent SECA with the same purpose as modified TECA, i.e., to maintain an erect ear carriage. However, this technique required comparatively less dissection and maintained a portion of the distal vertical ear canal as well. The cat had no complications during the 8-month follow-up period. The technique was eventually expanded to include dogs with pendulous ears. It should be noted that SECA cannot be employed in animals with conditions affecting the vertical ear canal, as this preserved portion may potentially lead to infection or recurrence.

Two cats with CGA were cured by partial vertical ear canal resection resulting in excellent aesthetic and functional outcomes as well as preservation of the external auditory canal [46]. This method is preferred when the vertical ear canal has neoplasia or hyperplasia, with a relatively normal horizontal ear canal [62] and is far simpler to perform compared to a complete vertical ear canal resection with less surgical trauma to the animal. One of the two cats developed a CGAc a year after the resection which was then resected successfully. It is indeed important to keep in mind that if the condition extends deeper into the vertical ear canal or beyond, conversion into a complete vertical ear canal resection/ablation or TECA would be necessary.

Following a video otoscopic evaluation and biopsy, remnants of the CGT were ablated with a CO_2_ laser (range: 10 to 20 watts, continuous wave mode; 30 s to 3 min) through the working channel of the video otoscope [31]. Limited post-operative complications were achieved with this method. Among the fourteen CGAc (four dogs, ten cats) and twelve CGA (seven dogs, five cats), otitis externa was noted in four dogs and recurrence in two animals (species unspecified; one CGA and CGAc, respectively). Similarly, Corriveau [48], reported the successful treatment of a feline CGA with CO_2_ laser (6–8 watts) ablation with no recurrence of lasered masses during the follow-up period of 2.5 years. Loft et al. [45] additionally treated 34 CGA-affected feline ears (23 cats) with a CO_2_ laser to yield minimal post-operative complications. However, a potential drawback of this technique was its inability to treat masses located beyond the external ear canal or in the deeper horizontal canal without damaging the normal tissues [31,45].

An approach wherein a complete cure was achieved without a TECA involved the insertion of a diode laser through the forceps channel of a video otoscope [25]. The complex CGA were excised with the laser followed by repeated cleansing of the ear canal and tympanic cavity using a video otoscope. The video-otoscopic therapy was carried out 14 times and complex CGA were excised by the diode laser thrice. Limitations of this mode included multiple sittings with repeated exposure to anesthesia and possible damage to regional nerves.

On the other hand, Takagi et al. [35] proposed HTH therapy as an alternative. It was performed at 45–65 °C for 10 min on day 0, day 28, and day 78 following which, the tumor disappeared on day 133 with no recurrence. However, similar to laser therapy, this is a suitable alternative only for superficial tumors.

A possible adjunct to surgery would be RT, although there are very few reports documenting its efficacy. Among the eleven animals (five dogs and six cats), four (two dogs and two cats with CGAc) had tumor recurrence (36%) and three (unspecified species with CGAc) had distant metastasis (27%) upon evaluation after RT [30]. Radiotherapy was found to be safe with minimal complications (acute reactions that resolved quickly). This study also highlighted the efficacy of megavoltage irradiation over orthovoltage in delivering radiation to deeper structures and thus reducing recurrence; but, treatment planning and delivery may be complicated due to the proximity of the tumor to the brain stem [23].

### 4.3. Diagnosis

Aural tumors are most often diagnosed when their mass effect manifests overt clinical signs [68]. Feline CGAc tend to exhibit more aggressive behavior than canine CGAc [9]. They also have metastatic potential [22,30,68], thus necessitating additional diagnostic procedures. Local invasion, especially with CGAc, is observed through the cartilage of the ear canal [68]. Although a high rate of metastasis isn’t typically reported with these tumors, up to 50% of them can metastasize to the regional lymph nodes, lungs, and viscera [13,22,28,53,54,55]. Metastasis to the regional lymph nodes was observed in 25% of cats on histopathology (unpublished cases). Seven dogs (63.63%, n = 7/11) and ten cats (83.33%, n = 10/12) were evaluated for evidence of pulmonary metastases. Suspicious pulmonary nodules were observed in a cat which died three days post-TECA-LBO, but no postmortem examination was performed. None of the dogs in the present study had radiographic evidence of pulmonary metastasis. Therefore, full staging including radiography and/or CT is advised as part of the diagnostic workup before treatment [68]. Additionally, prognostic factors such as MI/MF have been identified for CGAc [28,30,43].

Chronic inflammation with concurrent bacterial infection is considered one of the predisposing factors for this neoplasm [2,7]. Swabs from ear canals were cultured for bacteria in the majority of unpublished cases (63.63% of dogs and 50% of cats). At present, limited studies report bacterial isolates from ears affected with CGTs. Despite investigations, no association between bacteria and tumor development have been deduced [5].

Although CGA and CGAc are better determined via histopathology [42], cytology has the added advantage of an immediate tentative diagnosis indicating if the tumor is epithelial in origin with features of malignancy [2]. In a study on 27 feline ear canal tumors, 7 of 11 (64%) CGA/CGH and 6 of 7 (86%) CGAc diagnosed by histology were correctly diagnosed by cytology [42]. In the same study, inflammatory polyps and mast cell tumors were differentiated by cytology from CGA and CGAc, as well. This would hence facilitate traction avulsion or ventral bulla osteotomy for polyps whilst the others would require a more aggressive surgical approach [16]. However, cytology alone was unable to confirmatively diagnose CGTs in our study.

In all cases included in this review, histopathology was the most used method of diagnosis. Among the unpublished cases, mitotic count demonstrated no prognostic significance; however, it may hold relevance when a larger cohort of subjects are involved.

Although histopathology was the preferred mode of diagnosis in most cases, nuclear cytomorphometry could also be used as an auxiliary method to distinguish between canine ceruminous adenomas and adenocarcinomas [39]. The data obtained in his study also showed that quantitative nuclear cytomorphometric analysis has the potential to offer supplementary insights regarding the biological behavior of metastasizing canine CGAc. The research also suggested that morphometric variables may serve as valuable indicators for identifying tumors with an elevated risk of progression.

Alternative diagnostics used included ultrasonographic examination [41] and FNAC/FNAB/FNB [42]. An added advantage of ultrasonography was that it did not require sedation/anesthesia, unlike a biopsy, for example, from the ear canal. It was further used to examine the adjacent parotid glands to determine the extent of the CGT. Ceruminous gland adenomas manifested as regular, homogenously hypo-echoic solitary masses in the ear canal wall, whereas CGAc were irregular, lobulated masses with heterogeneous echogenicity. The local invasion of CGAc into the parotid gland was readily detected by ultrasonographic examination. This was evident as an enlarged, round hypo-echoic structure connected to the ear canal mass [41].

Despite the above-mentioned details, tumors arising from the ceruminous glands can present a diagnostic dilemma [69,70] due to their varied clinical and histological manifestations. Immunohistochemistry used in isolation or as a stand-alone test provides spurious results [57]. Therefore, histopathological examination with the addition of IHC has been suggested for efficient confirmatory diagnosis [71]. In this study, IHC was performed in five cases (four canine and one feline). Needless to say, the expression pattern of various biomarkers in canine and feline CGTs have not been well characterized highlighting the necessity for future research in this area.

### 4.4. Outcome

After successful excision of a CGA, the outlook is very promising. In cases of malignancy, there is a possibility of local recurrence or metastasis, but many animals still show positive progress after surgery. It was also observed that dogs with tumors restricted to either the vertical or horizontal ear canal experience better survival rates compared to those with tumors extending through the ear canal and bulla [3].

Median survival time of 878 days for dogs and 297 days for cats with CGAc was recorded. Dogs and cats with CGAc had a 6-month survival of 44.44% and 50% and a one-year survival of 44.44% and 25%, respectively. Cats with CGAc had a greater 6-month survival rate, but it declined by half at one year, suggesting that these tumors have poor long-term prognosis. In contrast, dogs showed a stable survival rate at 6 months and 1 year indicating a slower progression of the disease (unpublished cases).

The authors also observed that in contrast to four of eight dogs with squamous cell carcinoma only three of twenty-three dogs with CGAc died due to disease. Similarly, the median survival time of cats with CGAc was significantly greater than for cats with squamous cell carcinoma [13], thus, implying canine and feline aural squamous cell carcinomas probably exhibit a more aggressive biological nature with poor prognosis compared to CGAc.

### 4.5. Limitations

Despite the above-mentioned alternatives and their success, the results of the study must be considered within its limitations. The group of animals studied was small. A significant number of cases were lost to follow-up or died from other causes. Cases analyzed could be skewed towards a subset of dogs and cats whose owners were willing to pursue surgical intervention and histology thus missing other owners that declined further diagnosis. Limited information was available concerning all the objectives to provide a substantial and conclusive result. It is thus recommended to perform future prospective, multicenter studies to include more cases and explore possible genetic or inherited predisposition, clinical outcomes in terms of metastasis potential and long-term prognosis, the validity of alternative surgical approaches, innovative adjuvant therapies targeting metastasis, and residual tumor cells at post-surgical sites such as electrochemotherapy and intratumoral ablations as well as immunotherapy to gather more comprehensive information.

## 5. Conclusions

This study summarized the current literature available on clinical presentation, treatment outcome, survival, and biomarker studies on canine and feline CGTs. Although limited publications (17 canine and 19 feline) and unpublished cases were thoroughly analyzed, substantial data with special emphasis on treatment approaches were achieved from the same. The results also showed an increase in these cases in relation with chronic otitis either being recorded or diagnosed over the years thus emphasizing the requirement for larger, more advanced studies concerning adjunct treatment, diagnostics, and prognostic indicators especially for malignant tumors.

## Figures and Tables

**Table 1:** Signalment, clinical presentation, diagnosis, therapy, and outcome of the unpublished Malaysian CGT cases.

Case	Breed, Gender, Age (Years, Months)	Location	Cytology	Histology	Diagnostic Imaging		Surgical Management	Survival Outcome	Recurrence
					Radiography	CT			
**CANINE**									
1	ACS, IM, 6	Base and VEC	−	CGA	+	−	Unilateral TECA	LFU	+ (183 days PO)
2	Shih Tzu, IM, 10	VEC	+	CGAc	+	−	Unilateral TECA	Euthanized	−
3	Siberian Husky, CM, 7	VEC	−	CGAc	−	+	Unilateral TECA	LFU	−
4	GSD, IM, 12	Pinna, extending towards the canal	−	CGAc	+	−	Unilateral VECA	Died due to disease (770 days PO)	−
5	Local, IM, 8	Pinna, extending towards the canal	+	CGAc	−	+	Unilateral TECA-LBO	Died due to disease (668 days PO)	−
6	Rottweiler, IF, 7	Pinna, extending towards the canal	−	CGAc	−	+	Unilateral TECA-LBO	LFU	+ (19 days PO)
7	Poodle, IF, 7	VEC	−	CGAc	+	−	Unilateral TECA-LBO	Died (0-day PO)	−
8	Toy Poodle, CM, 11	HEC, extending towards the bulla	−	CGAc	−	+	Unilateral TECA-LBO	LFU	−
9	Local, IM, 14	Base and VEC	+	CGAc	−	−	Incisional biopsy	Died (424 days PO)	−
10	Shih Tzu, IM, 12	Base only	−	CGAc	−	−	Excisional biopsy	LFU	−
11	Shih Tzu, IM, 12	VEC	−	CGA	+	−	Unilateral TECA	Alive	−
**FELINE**									
1	Persian, IM, 6	Base and VEC	−	CGAc	+	−	Unilateral TECA	LFU	−
2	Persian, IF, 7	EAC	+	CGAc	+	+	Unilateral TECA-LBO *	Died due to disease (183 days PO)	+ (49 days PO)
3	MC, SF, 11,6	VEC	−	CGA	+	−	Traction and lumpectomy	Died (1974 days PO)	−
4	MC, CM, 8	Pinna, base and VEC	−	CGAc	+	−	Unilateral TECA-LBO	Died (369 days PO)	−
5	MC, CM, 8	VEC	−	CGA	−	+	Unilateral VBO and mass traction	LFU	−
6	Persian, IM, 2,7	VEC	−	CGA	+	−	Bilateral TECA-LBO	Died (63 days PO)	−
7	Persian, CM, 9	Base and VEC	+	CGAc	−	+	Unilateral TECA-LBO	Died due to disease (129 days PO)	+ (39 days PO)
8	DLH, CM, 6,10	Pinna, base and VEC	−	CGA	−	+	Unilateral TECA-LBO	Died (3 days PO)	−
9	Bengal, IM, 3,3	Base and VEC	−	CGAc	−	−	Bilateral TECA-LBO	LFU	−
10	DSH, SF, 8	EAC	+	CGAc	−	+	Unilateral TECA-LBO	Died due to disease (260 days PO)	+ (181 days PO)
11	AC, IM, 8	Pinna and VEC	−	CGAc	+	−	Incisional Biopsy	Alive	−
12	MC, CM, 7	Pinna and VEC	+	CGAc	+	+	Unilateral TECA-LBO	Alive	−

ACS: American Cocker Spaniel; GSD: German Shepherd; MC: Maine Coon; DSH: Domestic Short Hair; DLH: Domestic Long Hair; AC: American Curl; IM: Intact Male; CM: Castrated Male; IF: Intact Female; SF: Spayed Female; CT: Computed Tomography; VEC: Vertical Ear Canal; HEC: Horizontal Ear Canal; EAC: External Auditory Canal; CGA: Ceruminous Gland Adenoma; CGAc: Ceruminous Gland Adenocarcinoma; TECA: Total Ear Canal Ablation; TECA-LBO: Total Ear Canal Ablation And Lateral Bulla Osteotomy; VECA: Vertical Ear Canal Ablation; LFU: Lost To Follow-Up; PO: Post-operation; * Underwent Chemotherapy.

## Data Availability

Data are contained within the article. Additional datasets/Microsoft Excel sheets supporting the findings of this study are available from the corresponding author (G.T.S.) on request.

## Appendix A

**Table A1:** Studies on canine CGTs published between 1980 and 2023.

Continent	Country	Type of Publication	No. of Cases	Year of Publication	Cited by (Source: Scopus)	Reference
Asia	Japan	Case Report	1	2015	5	[25]
		Case Series	1	2014	3	[35]
	Korea	Case Report	1	2019	1	[32]
	India	Case Report	1	2023	0	[36]
	Japan	Article	1	2022	0	[38]
	India	Short Communication	1	2016	0	[37]
	Malaysia	Case Series	11	Unpublished	-	-
Europe	Italy	Case Report	1	2011	6	[33]
	Bulgaria	Article	16	2012	3	[39]
	Netherlands	Article	17	1986	38	[26]
	Portugal	Short Communication	6	2008	19	[27]
North America	USA	Case Series	62	1996	60	[28]
		Article	3	2015	9	[40]
		Article	-	2022	2	[29]
		Article	27	1996	92	[13]
		Retrospective Study	12	2013	24	[34]
		Article	9	1994	38	[30]
		Retrospective Study	11	2023	0	[31]

**Table A2:** Studies on feline CGTs published between 1980 and 2023.

Continent	Country	Type of Publication	No. of Cases	Year of Publication	Cited by (Source: Scopus)	Reference
Africa	Libya	Case Report	1	2018	1	[14]
	Egypt	Article	10	2021	0	[41]
Asia	Japan	Article	2	2017	5	[51]
	Malaysia	Case Series	12	Unpublished	-	-
Europe	Italy	Article	18	2005	31	[42]
	UK	Article	7	1992	28	[49]
		Article	18	2003	50	[43]
North America	USA	Case Series	62	1996	60	[28]
		Article	4	2004	12	[44]
		Article	-	2022	2	[29]
		Retrospective Study	25	1996	92	[13]
		Article	57	2022	4	[45]
		Case Series	2	2019	3	[46]
		Retrospective Study	4	2013	24	[34]
		Article	6	1994	38	[30]
		Article	15	2023	0	[31]
		Article	1	2006	15	[47]
		Article	11	1986	38	[26]
		Case Report	1	2012	11	[48]
		Case Report	1	2023	0	[50]

**Table A3:** Distribution of canine ceruminous gland tumors (CGTs) across the selected studies.

Sl.No	Ceruminous Gland Adenoma (CGA)	Ceruminous Gland Adenocarcinoma (CGAc)	Mixed Ceruminous Gland Tumor (CGT)	Total Number of Cases	Surgical Management	Reference
CANINE						
1	1 *	-		1	Excision using a diode laser	[25]
2	-	1		1	TECA, HTH	[35]
3	-	1		1	TECA	[32]
4	1	-		1	Excision	[36]
5	-	1		1	Resection	[38]
6	1	-		1	Excision	[37]
7	-	1		1	Euthanized, PME	[33]
8	8	8		16	Excision, Biopsy specimen	[39]
9	9 and 1 *	5	1 and 1 ^O^	17	Biopsy specimen	[26]
10	23	38	1	62	Biopsy specimen	[22]
11	-	3		3	Biopsy specimen	[40]
12	-	-		-	TECA-LBO	[29]
13	4	23		27	NS	[13]
14	-	2 ^s^ and 2 **	2 ^M^	6	Biopsy specimen	[27]
15	5	7		12	TECA-LBO	[34]
16	-	9		9	Resection, LECR, TECA, RT	[30]
17	4	7		11	Video-otoscopic aided biopsy and CO_2_ laser ablation	[31]
Unpublished	2	9		11	TECA, TECA-LBO, VECA, Traction, Biopsy specimens	-
Total	59	117	5	181		

* Complex CGA; ** Complex CGAc; ^s^ Simple CGAc. ^O^ Adenoma/adenocarcinoma; ^M^ Mixed CGAc; NS: Not specified. TECA: Total Ear Canal Ablation; HTH: High Temperature Hyperthermia; TECA-LBO: Total Ear Canal Ablation and Lateral Bulla Osteotomy; LECR: Lateral Ear Canal Resection; RT: Radiation therapy; VECA: Vertical Ear Canal Ablation; PME: Postmortem examination.

**Table A4:** Distribution of feline ceruminous gland tumors (CGTs) across the selected studies.

Sl.No	Ceruminous Gland Adenoma (CGA)	Ceruminous Gland Adenocarcinoma (CGAc)	Mixed Ceruminous Gland Tumor (CGT)	Total Number of Cases	Surgical Management	Reference
FELINE						
1	-	2		2	Biopsy specimen	[51]
2	-	1		1	Resection	[14]
3	3	7		10	TECA	[41]
4	11	7		18	Biopsy specimen	[42]
5	-	7		7	TECA-LBO	[49]
6	-	18		18	TECA	[43]
7	19	43		62	Biopsy specimen	[28]
8	2	2		4	Modified TECA	[44]
9	-	-		_	TECA-LBO	[23]
10	3	22		25	NS	[13]
11	57	-		57	Biopsy specimen, TECA-LBO, pinnectomy, CO_2_ laser therapy	[45]
12	2	-		2	Partial vertical ear canal resection	[46]
13	3	1		4	TECA-LBO	[34]
14	-	9		9	Resection, LECR, TECA, RT	[30]
15	10	5		15	Video-otoscopic aided biopsy and CO_2_ laser ablation	[31]
16	1	-		1	Subtotal ear canal ablation	[47]
17	8	3		11	Biopsy specimen	[26]
18	1	-		1	CO_2_ laser ablation	[48]
19	-	1		1	Exploratory surgery of the ear canal with LBO	[50]
Unpublished	4	8		12	TECA, TECA-LBO, VBO, Traction, Biopsy specimens	-
Total	124	136		260		

TECA: Total Ear Canal Ablation; TECA-LBO: Total Ear Canal Ablation and Lateral Bulla Osteotomy; LECR: Lateral Ear Canal Resection; LBO: Lateral Bulla Osteotomy; RT: Radiation therapy; VBO: Vertical Bulla Osteotomy.
